# Correction: Overall survival of patients with KIT-mutant metastatic GIST in the era of multiple kinase inhibitor availability

**DOI:** 10.1007/s00432-024-06056-y

**Published:** 2025-01-11

**Authors:** Valerie Haller, Carina Reiff, Rainer Hamacher, Karina Kostbade, Moritz Kaths, Juergen Treckmann, Stefanie Bertram, Yasmin Zaun, Sebastian Bauer, Johanna Falkenhorst

**Affiliations:** 1 Department of Medical Oncology and Sarcoma Center, West German Cancer Center, University Hospital Essen, Essen, Germany; 2 German Cancer Consortium (DKTK), Partner Site University Hospital Essen, Essen, Germany; 3 Department of Visceral and Transplantation Surgery, University Hospital Essen, Essen, Germany; 4 Department of Pathology, University Hospital Essen, Essen, Germany

**Correction to: Journal of Cancer Research and Clinical Oncology (2024) 150:489**

10.1007/s00432-024-05965-2

In Table 1 of this article, row labels and their corresponding data were misaligned during the typesetting process. The correct alignment is now present; the data itself remains unaffected.

**Table 1:** Description of demographics

**Gender**	*n* = 174
• Male	*n* = 103 (59%)
• Female	*n* = 71 (41%)
**Follow-up**	*n* = 174
• Alive	*n* = 97 (56%)
• Dead	*n* = 77 (44%)
o Tumor-related death	*n* = 71 (92.2%)
o No tumor- related death	*n* = 6 (7.8%)
▪ Cardio-vascular events	*n* = 4 (66.8%)
▪ Unknown, but regressive tumor findings under continued therapy	*n* = 1 (16.6%)
▪ Local complication under non-GIST-associated secondary tumor (cervical squamous cell carcinoma)	*n* = 1 (16.6%)
**Age at time of diagnosis** (years) (*n* = 174)	Median 57 (27–80)
**Age at time of diagnosis of metastases** (years) (*n* = 174)	Median 59 (27–82)
**Year of diagnosis of metastases** (*n* = 174)	2008–2021
**Follow up since diagnosis (years)** (*n* = 174)	Median 6.6 years
• Minimum	1 year
• Maximum	25.5 years
**Follow up since diagnosis of metastases** (years) (*n* = 174)	Median 4.7 years
• Minimum	0.1 year
• Maximum	14.5 years
**Follow up since first appointment in patients with metastatic disease** (years) (*n* = 174)	Median 2.54 years
• Minimum	0 months
• Maximum	14.2 years
**Primary mutations**	*n* = 174
• KIT exon 11	*n* = 138 (79%)
• KIT exon 9	*n* = 36 (21%)
**Location of primary**	*n* = 174
• Gastric	*n* = 60 (34.5%)
• Small intestine	*n* = 93 (53.5%)
• Colon	*n* = 9 (5%)
• Other (not specified)	*n* = 12 (7%)
**Location of metastases**	*n* = 174
• Peritoneum	*n* = 67 (38.5%)
• Liver	*n* = 64 (36.8%)
• Liver and Peritoneum	*n* = 40 (23%)
• Other (pancreas, small intestine)	*n* = 3 (1.7%)
**Time to diagnosis of metastases**	*n* = 174
• Metachronous	*n* = 85 (48.9%)
• Synchronous	*n* = 89 (51.1%)
**Time to diagnosis of metastases** (in months)	Median 5 (0-210 months)
**Resection of primary**	*n* = 174
• Yes	*n* = 146 (84%)
• No	*n* = 28 (16%)
**Metastasectomy**	*n* = 174
• Yes	*n* = 96 (55.2%)
• Location of metastases	
o Liver	*n* = 35 (36.5%)
o Peritoneum	*n* = 38 (39.6%)
o Liver and peritoneum	*n* = 22 (22.9%)
o Missing	*n* = 1 (1%)
• No/TKI-treatment only	*n* = 78 (44.8%)
**Perioperative imatinib therapy for initial localized disease**	*n* = 75
o Excluded patients (resulting in *n* = 75 patients analyzed for perioperative imatinib therapy for initial localized disease)	
▪ Patients who received adjuvant imatinib but discontinued treatment after less than 5 months due to toxicities	*n* = 7
▪ Patients who received additive therapy after an initial tumor rupture	*n* = 3
• Perioperative imatinib treatment	*n* = 48 (64%)
o Relapse Risk in localized disease	
▪ 0–40%	*n* = 4 (8.3%)
▪ 40–60%	*n* = 5 (10.4%)
▪ 60–80%	*n* = 8 (16.7%)
▪ 80–90%	*n* = 7 (14.6%)
▪ 90–100%	*n* = 13 (27.1%)
▪ Missing	*n* = 11 (22.9%)
• No perioperative imatinib treatment	*n* = 27 (36%)
o Relapse Risk in localized disease	
▪ 0–40%	*n* = 7 (25.9%)
▪ 40–60%	*n* = 3 (11.1%)
▪ 60–80%	*n* = 1 (3.7%)
▪ 80–90%	*n* = 2 (7.4%)
▪ 90–100%	*n* = 5 (18.5%)
▪ Missing	*n* = 9 (33.4%)
• Reason for no perioperative imatinib therapy	
o Treatment not offered	*n* = 25 (92.6%)
o Patient refused treatment	*n* = 1 (3.7%)
o Unknown	*n* = 1 (3.7%)
**Time between initial diagnosis and first appointment** (in months) (*n* = 172)	Median 27 (0-290 months)
**Place for primary treatment**	*n* = 174
• Local hospital	*n* = 153 (87.9%)
• Sarcoma center	*n* = 12 (6.9%)
• Practicing oncologist	*n* = 9 (5.2%)
**Place of treatment for metastatic disease**	*n* = 174
• Local hospital	*n* = 102 (58.6%)
• Sarcoma center	*n* = 64 (36.8%)
• Practicing oncologist	*n* = 8 (4.6%)
**State of disease at first appointment**	*n* = 174
• First diagnosis of metastatic disease	*n* = 32 (18.4%)
• External diagnosis of metastatic disease	*n* = 142 (81.6%)
**Palliative treatment lines** (*n* = 174)	Median 4 (1–8)
**Number of treatment lines in patients who died of their disease**	*n* = 71
• Range	1–8
• Median	4
o 1 treatment line	*n* = 2 (2.8%) (cum. 100%)
o 2 treatment lines	*n* = 6 (8.5%) (cum. 97.2%)
o 3 treatment lines	*n* = 7 (9.8%) (cum. 88.7%)
o 4 treatment lines	*n* = 22 (31%) (cum. 78.9%)
o 5 treatment lines	*n* = 19 (26.8%) (cum. 47.9%)
o >=6 treatment lines	*n* = 15 (21.1%) (cum. 21.1%)
**Number of treatment lines in patients with no tumor-related death**	*n* = 6
• Range	1–5
• Median	2
o 1 treatment line	*n* = 2 (33.3%)
o 2 treatment lines	*n* = 2 (33.3%)
o 4 treatment lines	*n* = 1 (16.7%)
o 5 treatment lines	*n* = 1 (16.7%)
**Medication as part of clinical trial at any point during treatment**	*n* = 174
• Yes	*n* = 50 (28.7%)
o Patients with tumor-related death	*n* = 33 (66%)
• No	*n* = 124 (71.3%)

The incorrect alignment of Table 1 is shown below:

**Table 1:** Description of demographics

**Gender**	n = 174
Male	n = 103 (59%)
Female	n = 71 (41%)
**Follow-up**	n = 174
Alive	n = 97 (56%)
Dead	n = 77 (44%)
Tumor-related death	n = 71 (92.2%)
• No tumor-related death	n = 6 (7.8%)
• Cardio-vascular events	n = 4 (66.8%)
• Unknown, but regressive tumor findings under continued therapy	n = 1 (16.6%)
• Local complication under non-GIST-associated secondary tumor (cervical squamous cell carcinoma)	n = 1 (16.6%)
**Age at time of diagnosis** (years) (n = 174)	Median 57 (27–80)
**Age at time of diagnosis of metastases** (years) (n = 174)	Median 59 (27–82)
**Year of diagnosis of metastases** (n = 174)	2008–2021
**Follow up since diagnosis (years)** (n = 174)	Median 6.6 years
Minimum	1 year
Maximum	25.5 years
**Follow up since diagnosis of metastases** (years) (n = 174)	Median 4.7 years
Minimum	0.1 year
Maximum	14.5 years
**Follow up since first appointment in patients with metastatic disease** (years) (n = 174)	Median 2.54 years
Minimum	0 months
Maximum	14.2 years
**Primary mutations**	n = 174
KIT exon 11	n = 138 (79%)
KIT exon 9	n = 36 (21%)
**Location of primary**	n = 174
Gastric	n = 60 (34.5%)
Small intestine	n = 93 (53.5%)
Colon	n = 9 (5%)
Other (not specified)	n = 12 (7%)
**Location of metastases**	n = 174
Peritoneum	n = 67 (38.5%)
Liver	n = 64 (36.8%)
Liver and Peritoneum	n = 40 (23%)
Other (pancreas, small intestine)	n = 3 (1.7%)
**Time to diagnosis of metastases**	n = 174
Metachronous	n = 85 (48.9%)
Synchronous	n = 89 (51.1%)
**Time to diagnosis of metastases** (in months)	Median 5 (0-210 months)
**Resection of primary**	n = 174
Yes	n = 146 (84%)
No	n = 28 (16%)
**Metastasectomy**	n = 174
Yes	n = 96 (55.2%)
• Location of metastases	
• Liver	n = 35 (36.5%)
• Peritoneum	n = 38 (39.6%)
• Liver and peritoneum	n = 22 (22.9%)
• Missing	n = 1 (1%)
No/TKI-treatment only	n = 78 (44.8%)
**Perioperative imatinib therapy for initial localized disease**	n = 75
• Excluded patients (resulting in n = 75 patients analyzed for perioperative imatinib therapy for initial localized disease)	
• Patients who received adjuvant imatinib but discontinued treatment after less than 5 months due to toxicities	n = 7
• Patients who received additive therapy after an initial tumor rupture	n = 3
Perioperative imatinib treatment	n = 48 (64%)
• Relapse Risk in localized disease	
• 0–40%	n = 4 (8.3%)
• 40–60%	n = 5 (10.4%)
• 60–80%	n = 8 (16.7%)
• 80–90%	n = 7 (14.6%)
• 90–100%	n = 13 (27.1%)
• Missing	n = 11 (22.9%)
No perioperative imatinib treatment	n = 27 (36%)
• Relapse Risk in localized disease	
▪ 0–40%	n = 7 (25.9%)
• 40–60%	n = 3 (11.1%)
• 60–80%	n = 1 (3.7%)
• 80–90%	n = 2 (7.4%)
• 90–100%	n = 5 (18.5%)
• Missing	n = 9 (33.4%)
• Reason for no perioperative imatinib therapy	
• Treatment not offered	n = 25 (92.6%)
• Patient refused treatment	n = 1 (3.7%)
• Unknown	n = 1 (3.7%)
**Time between initial diagnosis and first appointment** (in months) (n = 172)	Median 27 (0-290 months)
**Place for primary treatment**	n = 174
• Local hospital	n = 153 (87.9%)
Sarcoma center	n = 12 (6.9%)
Practicing oncologist	n = 9 (5.2%)
**Place of treatment for metastatic disease**	n = 174
Local hospital	n = 102 (58.6%)
Sarcoma center	n = 64 (36.8%)
Practicing oncologist	n = 8 (4.6%)
**State of disease at first appointment**	n = 174
First diagnosis of metastatic disease	n = 32 (18.4%)
External diagnosis of metastatic disease	n = 142 (81.6%)
**Palliative treatment lines** (n = 174)	Median 4 (1–8)
**Number of treatment lines in patients who died of their disease**	n = 71
Range	1–8
Median	4
• 1 treatment line	n = 2 (2.8%) (cum. 100%)
• 2 treatment lines	n = 6 (8.5%) (cum. 97.2%)
• 3 treatment lines	n = 7 (9.8%) (cum. 88.7%)
• 4 treatment lines	n = 22 (31%) (cum. 78.9%)
• 5 treatment lines	n = 19 (26.8%) (cum. 47.9%)
• >=6 treatment lines	n = 15 (21.1%) (cum. 21.1%)
**Number of treatment lines in patients with no tumor-related death**	n = 6
Range	1–5
Median	2
• 1 treatment line	n = 2 (33.3%)
• 2 treatment lines	n = 2 (33.3%)
• 4 treatment lines	n = 1 (16.7%)
• 5 treatment lines	n = 1 (16.7%)
**Medication as part of clinical trial at any point during treatment**	n = 174
Yes	n = 50 (28.7%)
• Patients with tumor-related death	n = 33 (66%)
No	n = 124 (71.3%)

The original article has been corrected.

